# Chronic asymptomatic orchitis in dogs alters Sertoli cell number and maturation status

**DOI:** 10.3389/fvets.2025.1519105

**Published:** 2025-02-05

**Authors:** Pauline Rehder, Eva-Maria Packeiser, Hanna Körber, Sandra Goericke-Pesch

**Affiliations:** Reproductive Unit – Clinic for Small Animals, University of Veterinary Medicine Hannover, Foundation, Hannover, Germany

**Keywords:** AMH, CK18, Sertoli cell number, chronic asymptomatic orchitis, infertility, dog

## Abstract

Infertility due to non-obstructive azoospermia is a common diagnosis in infertile male dogs. Chronic asymptomatic orchitis (CAO) has been postulated as a significant cause of non-obstructive azoospermia in acquired male canine infertility. Despite severe microenvironmental changes, some resilient spermatogonial stem cells persist in CAO-affected testes. As Sertoli cells play an essential role in spermatogenesis and the testicular micromilieu, they represent a new target for CAO potential treatment and consequently deserve further investigation. To investigate Sertoli cell number and maturational status, different markers [Vimentin, anti-Müllerian hormone (AMH), and cytokeratin-18 (CK18)] were evaluated in healthy and CAO-affected testes at mRNA and protein levels. Sertoli cell number was reduced in CAO-affected dogs. Sertoli cells also partly returned to an immature status, as indicated by the expression of AMH and CK18 at mRNA and protein levels. The degree of spermatogenesis disruption matched with the degree of Sertoli cell alterations. The investigation of CAO in this study is limited by the number of samples and the lack of testicular volume measurements, but this does not diminish its importance in new findings. In conclusion, this study identifies alterations in Sertoli cell number and maturation status as a cause or consequence of CAO. The results indicate the need to restore Sertoli cell function as a potential therapeutic target for a successful restart of spermatogenesis.

## 1 Introduction

Acquired infertility in male dogs represents a bitter emotional and financial loss for the breeders, especially as it is often irreversible. It is usually characterized by azoospermia, the absence of sperm in the ejaculate, most often of non-obstructive nature [non-obstructive azoospermia (NOA)] (1, 2). NOA is not only a problem that plays a role in dogs, but it also affects approximately 10% of all infertile men. There is currently no treatment available, in human and veterinary medicine (3–5). Despite this, little is known about the underlying etiology or associated histological changes in the testicular tissues of affected dogs with acquired infertility. Recently, we characterized for the first time a larger collective of dogs with NOA histologically. In addition to disrupted spermatogenesis at various levels, including Sertoli cell-only (SCO) syndrome, we identified fibrosis and significant immune cell infiltration of lymphoplasmacytic character (6). The findings resembled those of autoimmune orchitis previously described as individual cases in dogs (7–12). Due to the lack of clinical symptoms of bacteriological and endocrine causes, we instead defined our findings as chronic asymptomatic immune-mediated orchitis (CAO) (1). Based on the frequency in our random study population (>90%), we defined CAO as the most common cause of acquired infertility in male dogs (1). Continuous inflammation plays a vital role in CAO as indicated by prostaglandin endoperoxidase synthase 2 [PTGS2, formerly cyclooxygenase-2 (COX-2)] overexpression (13, 14), significantly altering the spermatogonial stem cell niche. Germ cells are affected considerably by apoptosis in CAO-affected testes (14), resulting not only in an overall loss of germ cells but also in a significant reduction of spermatogonial stem cells, SSCs (15), reducing the chances for recovery of testicular function. On the contrary, the Sertoli cells (SCs) appeared to be unaffected or less affected by apoptosis, possibly related to the increased expression of the anti-apoptotic factor Bcl-2 (16).

It is well known that SCs play an essential role in physiological spermatogenesis as they support germ cells—structurally and functionally (17–22). They are the only somatic cells within the seminiferous tubules and form the structure of the germinal epithelium. SCs reach from the basal membrane to the lumen of the seminiferous tubules and surround the different generations of germ cells with their cytoplasm. Independent of species, age, and maturation status, they express vimentin, an intermediate filament protein (23–29). SCs form the blood–testis barrier; its integrity is crucial for the normal development of germ cells. They determine the destiny and fate of germ cells by nourishing and supporting them as well as influencing the testicular micromilieu (18, 21). Interestingly, we identified apparent morphological changes in SCs, namely, round or ovoid nuclei and a more adluminal localization in canine CAO-affected testicular samples, putting them into the focus of interest (1). It appears probable that alterations in SC number, structure, and function are related to modifications of the testicular microenvironment in canine CAO as postulated in human NOA patients (29, 30). Trying to clarify the underlying etiology, Zhao et al. (30) postulated the relevance of the SC maturation status with SCs in human idiopathic NOA being physiologically immature, but SCs in congenital NOA being mature. Currently, information about the maturation state of SCs in canine CAO is lacking. Regarding the numerical changes of SCs, a reduction in SC number would influence the fate of SSCs as there occurs to be a fixed germ cell–SC ratio (22, 31, 32).

While Vimentin is expressed through all states of SC maturation, anti-Mullerian hormone (AMH) and (CK18) are known SC immaturity markers in dogs (33, 34), rats (35), horses (36), and humans (32, 37, 38). AMH, initially recognized for its role in Müllerian duct regression during male fetal development (39, 40), is known to be secreted explicitly by SCs in the neonatal testes (33, 41). On the contrary, adult SCs usually do not express AMH. Re-expression of AMH in canine and human SCs is associated with testicular atrophy, SC tumors, and in response to GnRH-agonist treatment, indicating a return to an immature SC phenotype (42–46). CK18 is an intermediate filament protein expressed by epithelial cells, including fetal SCs, and is described to be absent in humans after the 20th gestational week (38). Just like AMH, CK18 is known to re-occur in various testis pathologies in adulthood, such as SCO, testis atrophy (42, 47–49), and cryptorchism in dogs (50), humans (32, 51, 52), and monkeys (53). Despite alterations in SC maturation status in canine CAO appear to be probable, it has never been investigated yet. Nevertheless, it remains to be clarified whether the re-expression of SC immaturity markers in specific testicular pathologies is a cause or consequence. However, understanding SC development and function in different pathologies is crucial for elucidating the mechanisms underlying male reproductive health and fertility and, thus, necessary to enable future therapeutic approaches.

To gain further insights into the role of SCs in canine CAO, we investigated whether the number of SCs is affected by CAO and whether their maturation status is altered as indicated by the re-expression of AMH and CK18. We hypothesize that our results comparing CAO-affected testes to juvenile testes and adult testes with normal spermatogenesis contribute to understanding why the CAO-SCs cannot support re-initiation of spermatogenesis.

## 2 Materials and methods

### 2.1 Study populations and tissue collection

Twenty-five clinically healthy and sexually mature and three juvenile dogs were included in this study. All testicular tissue samples were obtained from dogs castrated on owners’ request for other than medical reasons [control group (CG), *n* = 10, juvenile group (JG), *n* = 3] or for diagnostic purposes in the azoospermic dogs (*n* = 15). The CG (*n* = 10) consisted of healthy, normospermic dogs with a mean age of 3.8 ± 3.0 years (0.9–9.9 years) and belonging to the following breeds: Boston Terrier, Boxer, Chihuahua, Havanese, Maltese (*n* = 1 each), and Beagle (*n* = 3) or were crossbreeds (*n* = 2). The age of juvenile, mongrel dogs (*n* = 3) was 8 weeks. The azoospermic dogs (*n* = 15) were presented at the clinic for semen analysis for infertility reasons. They underwent detailed clinical andrological, sonographical, endocrine, and microbiological examinations to rule out clinical disease, testicular tumor, obstructive azoospermia, as well as endocrine and microbiological pathologies. As described in a previous study (1), all dogs were diagnosed with CAO as defined by significant immune cell infiltration and disruption of spermatogenesis. CAO dogs had a mean age of 5.5 ± 1.9 years (2.5–9.5 years). They included the following breeds: Beagle, Cairn Terrier, Cane Corso, Coton de Tulear, Iceland Sheepdog, Jack Russel Terrier, Labrador Retriever, Miniature Poodle, Welsh Corgi Pembroke (*n* = 1 each), Collie, and German Shepherd (*n* = 3 each).

According to the histological findings, this group was divided into two subgroups: 1. “early arrest,” including SCO or spermatogenesis arrested at the level of spermatogonia (*n* = 6); and 2. “late arrest,” with spermatogenesis being arrested at the level of spermatocytes or later stages (*n* = 9). All testicular tissue samples were processed as described in previous studies (13, 54, 55). Due to the lack of freshly frozen tissue, the juveniles could only be analyzed at the protein level in the immunohistochemistry (IHC).

### 2.2 Quantitative real-time PCR

Preparation and analysis of RNA samples and cDNA were performed as described in a previous study (15). In brief, RNA was isolated, concentration and quality were measured, and cDNA was synthesized. Primer sets for RT-qPCR to measure the expression of *AMH* and *CK18* were drafted using known sequences available from GenBank (Table 1). *Glyceraldehyde-3 phosphatase dehydrogenase* (*GAPDH*) and *hypoxanthine guanine phosphoribosyltransferase* (*HPRT*) served as reference genes for endogenous controls.

**Table 1:** Sequences of primers for RT-PCR and RT-qPCR, amplicon length, efficiency, and accession number.

Primer	Oligonucleotide sequence (5′-3′)	Amplicon length (bp)	Efficiency	Accession number
*GAPDH*		228	2.05	NM_001003142
Forward	GGCCAAGAGGGTCATCATCTC			
Reverse	GGGGCCGTCCACGGTCTTCT			
*HPRT*		94	2.05	NM_001003357.2
Forward	TGACACTGGGAAAACAATGCA			
Reverse	GGTCCTTTTCACCAGCAAGCT			
*AMH*		86	2.03	NM_001314127.1
Forward	CTGCACCTGGAGGAAGTGACA			
Reverse	AGCTCTAGGGGACTGGCTCC			
*CK18*		188	2.14	NM_001346040.1
Forward	CAGTCCGTGGAGAGCGACAT			
Reverse	TCCAACTCCACCGTCAACCC			

Relative mRNA expression [quantitative reverse transcription polymerase chain reaction (RT-qPCR)] was analyzed as described in previous studies (56, 57): 2 μL of 1:10 diluted cDNA was added to 5 μL of FastStart Essential DNA Green Master (Roche Diagnostics GmbH, Mannheim, Germany), 1 μL of the forward and reverse primer (10 pmol, Microsynth AG, Balgach, Switzerland) (Table 1), and 1 μL of nuclease-free water. RT-qPCR was performed with the following cycling conditions for all genes: 95°C for 10 min, followed by 45 cycles of 95°C for 10 s, 60°C for 10 s, 72°C for 10 s, and melting curve. All samples were run in triplicates using a LightCycler^®^ real-time PCR system (software version 1.1.0.1320, Roche Diagnostics GmbH, Mannheim, Germany). Using a relative standard curve derived from a triplet RT-qPCR run of a 2-fold dilution series (1:2–1:128) of pooled cDNA samples, the PCR efficiencies of target and reference genes were calculated, whereas the efficiency (E) was calculated as E = 10(−1/*m*), where *m* is the slope of the linear regression line. For the evaluation of the RT-qPCR results, the efficiency-corrected relative quantification as described in a previous study by Pfaffl (58) was modified and extended taking both reference genes—*GAPDH* (59) and *HPRT* (15)—into account as described in a previous study (60). The specificity of the primers for *AMH* and *CK18* was confirmed by sequencing of PCR products (Microsynth AG) and by using BLAST.^1^

### 2.3 Immunohistochemistry

The IHC was performed as described in a previous study (59). The samples were sectioned at a thickness of 3 μm, deparaffinized in xylene, and rehydrated in a descending ethanol series. Antigen retrieval was performed by cooking in a microwave oven (Vimentin, CK18) or water bath (AMH) at 96°C for 15 min in citrate buffer (pH 6.0). After blocking the endogenous peroxidase reactivity by 3% hydrogen peroxide in methanol, unspecific binding was blocked with 10% horse serum (S-2000, Vector Laboratories, Newark, CA, United States) diluted in 3% bovine serum albumin (BSA, VWR Life Science, Solon, OH, United States). The sections were incubated with the corresponding primary antibody for 20 h at 4°C (Table 1). For negative controls, only ICC buffer (1.2 g Na_2_HPO_4_, 0.2 g KH_2_PO_4_, 0.2 g KCl, 8.0 g NaCl, 3-mL Triton X ad 1,000 mL) was used; host-matched irrelevant immunoglobulin G (IgG) in the same protein concentration as the primary antibodies served as isotype control (I-2000, Mouse IgG, Control Antibody, Vector Laboratories, Newark, CA, USA). ICC buffer was used for washing; then the Vimentin and AMH sections were incubated with a biotinylated horse anti-mouse secondary antibody (BA-2000, Vector Laboratories, dilution 1:100 for AMH, 1:200 for Vimentin) at room temperature for 30 min. Detection of immunoreactivity was performed via avidin/biotinylated peroxidase complex (VECTASTAIN PK-6101 Elite ABC Kit, Vector Laboratories, Newark, CA, USA) and NovaRED^®^ (Vector Nova-RED Substrate Kit SK-4800, Vector Laboratories, Newark, CA, USA). For CK18, detection of the immunopositive signal was performed by incubating the slides with SuperVision-2 HRP Enhancer (DCS, Innovative Diagnostic Systeme, Hamburg, Germany) and HRP Polymer (DCS, Innovative Diagnostic Systeme, Hamburg, Germany) for 20 min each. The immunopositive signal was visualized via DAB (DCS, Innovative Diagnostic Systeme, Hamburg, Germany). Afterwards, all slides were counterstained with Meyer’s hematoxylin. The last step was dehydrating the sections with ascending ethanol series and xylene followed by mounting with Roti^®^ Histokitt II (Roth AG, Arlesheim, Switzerland). Details about the used antibodies are shown in Table 2.

**Table 2:** Primary antibodies and dilutions used for immunohistochemistry.

Antibody	Stock keeping unit (SKU)	Host, clonality	Concentration (μg/mL)	Dilution	Isotype control	Secondary antibody	Detection kit
AMH	Sc-166752^a^	Mouse monoclonal	2	1:100	Mouse IgG	Horse Anti-Mouse IgG	Nova-RED
CK18	Progen 690028S^b^	Mouse monoclonal	2.5	1:20	Mouse IgG	DCS Super Vision 2^d^	DAB
Vimentin	Dako M0725^c^	Mouse monoclonal	0.312	1:500	Mouse IgG	Horse Anti-Mouse IgG	Nova-RED

a Santa Cruz Biotechnology, Dallas, TX, United States.

b PROGEN Biotechnik GmbH, Heidelberg, Germany.

c Dako, Agilent, Santa Clara, CA, United States.

d PD000KIT, DCS Innovative Diagnostik-Systeme, Hamburg, Germany.

Evaluation of the IHC staining was performed by using an Olympus BX41TF Microscope (Olympus^®^, Tokyo, Japan) with an Olympus DP72 camera (Olympus Corporation, Tokyo, Japan) and the Olympus cellSense Dimension Software (version 2.1, Olympus Corporation, Tokyo, Japan). All Vimentin-positive SCs with visible nucleus per tubule were counted in 20 nearly round tubules (CAO: 10 from each side) at 400-fold magnification to assess the total SC number. For AMH, 40 approximately round tubules of each dog (CAO: 20 of the left and right testis) were evaluated at 200-fold magnification with a semiquantitative ranking (0–3) of the staining intensity. For CK18, all positive SCs in the 40 approximately round tubules of each dog (CAO: 20 of the left and right testis) were counted.

### 2.4 Western blot

Western blot analysis was performed to verify the specificity of the primary AMH and CK18 antibodies used in the IHC. Details about the antibodies, dilutions/protein concentrations, and positive controls used are found in Table 3. The specificity of the monoclonal anti-vimentin antibody was established in earlier studies (23–25, 61).

**Table 3:** Overview of reagents, dilutions, and positive controls used in the Western blot.

Antibody	Stock keeping unit (SKU)	Concentration (μg/mL)	Dilution	Positive control	Isotype control	Secondary antibody	Dilution secondary antibody
AMH	Sc-166752^a^	2	1:100	HeLa	Mouse IgG	Horse Anti-Mouse IgG	1:1000
CK18	Progen 690028S^b^	0.167	1:300	MCF-7	Mouse IgG	Horse Anti-Mouse IgG	1:1000

a Santa Cruz Biotechnology, Dallas, TX, United States.

b PROGEN Biotechnik GmbH, Heidelberg, Germany.

Precision Plus Protein™ All Blue Prestained Protein Standards (Bio-Rad Laboratories, Hercules, CA, United States) was used as a ladder to identify the band size. In brief, gel electrophoresis was used to separate the protein of tissue and cell lysates on 4–20% gradient sodium dodecyl sulfate-polyacrylamide gel (Mini-Protean^®^ TGX™ Gels, Bio-Rad Laboratories, Hercules, CA, USA). Afterwards, the protein was transferred onto a PVDF membrane via Trans-Blot^®^ Turbo™ Transfer Pack (#1704156, Bio-Rad Laboratories). Blocking of unspecific signals was performed by incubating for 5 min with a blocking buffer (EveryBlot Blocking Buffer, Bio-Rad Laboratories, Hercules, CA, USA). The membranes were incubated overnight at 4°C with the primary antibody. The next day, after washing with TBST (tris-buffered saline, 0.1% Tween 20), a secondary antibody (biotinylated horse anti-mouse IgG antibody, BA-2000, Vector Laboratories, Newark, CA, USA) was added for 1 h. Signals were visualized by using Clarity™Western ECL Blotting Substrate (Bio-Rad Laboratories, Hercules, CA, USA) for 1 min. Subsequently, images were taken with ChemiDoc™ Imaging Systems with Image Lab™ Touch Software (Image Lab 6.0.1, Bio-Rad Laboratories, Hercules, CA, USA).

### 2.5 Statistical analysis

GraphPad Prism10 software (GraphPad Software, Inc., La Jolla, CA, United States) and Microsoft Excel (Version 16.81, Microsoft, Redmond, WA, United States) were used for statistical analysis. Values at a level of *p* < 0.05 were regarded as statistically significant. Our study aimed to identify significant differences between CAO, JG, and CG regarding their SC number, AMH, and CK18 mRNA and protein expressions. In a separate analysis, CAO was further differentiated into its subgroups early arrest (*n* = 6) and late arrest (*n* = 9) of spermatogenesis and the results compared to CG.

The mRNA expression ratios were initially tested for normal distribution by the Shapiro–Wilk test. As the data were log-normally distributed, *CK18* and *AMH* ratios were log-transformed and analyzed by ordinary one-way analysis of variance (ANOVA) followed by Tukey’s multiple comparison tests if *p* < 0.05 for comparisons of early arrest, late arrest and CG. An unpaired *t*-test was performed for comparisons between CAO and CG. The ratios (mRNA expressions) are presented as Box and Whisker Plot.

Similarly, the Shapiro–Wilk test was performed to confirm the normal distribution of SC number and AMH protein expression. A paired *t*-test was applied to prove whether the respective data differed depending on the localization in the CAO group (right/left testis). As no significant difference was observed, the results obtained from both testes of each dog were summarized, and the results were still normally distributed. Ordinary one-way ANOVA was applied, followed by Tukey’s multiple comparison tests if *p* < 0.05 to identify differences between early arrest, late arrest, JG, and CG as for overall group comparisons (CAO vs. CG vs. JG). The results for CK18 protein expression were not normally distributed. Consequently, the Wilcoxon test was applied to prove that there was no significant difference between the right and left testis, and the results were combined, with summarized data—still the results were not being normally distributed. Thereafter, non-parametric ANOVA (Kruskal–Wallis test) was performed for overall group comparison and more detailed comparisons with CAO divided into subgroups followed by Dunn’s multiple comparison test if *p* < 0.05. All protein expression data were presented as the arithmetic mean and standard deviation ( $\bar{x\;}$ ± SD) for comparative reasons.

## 3 Results

### 3.1 Sertoli cell number

**Figure 1:**
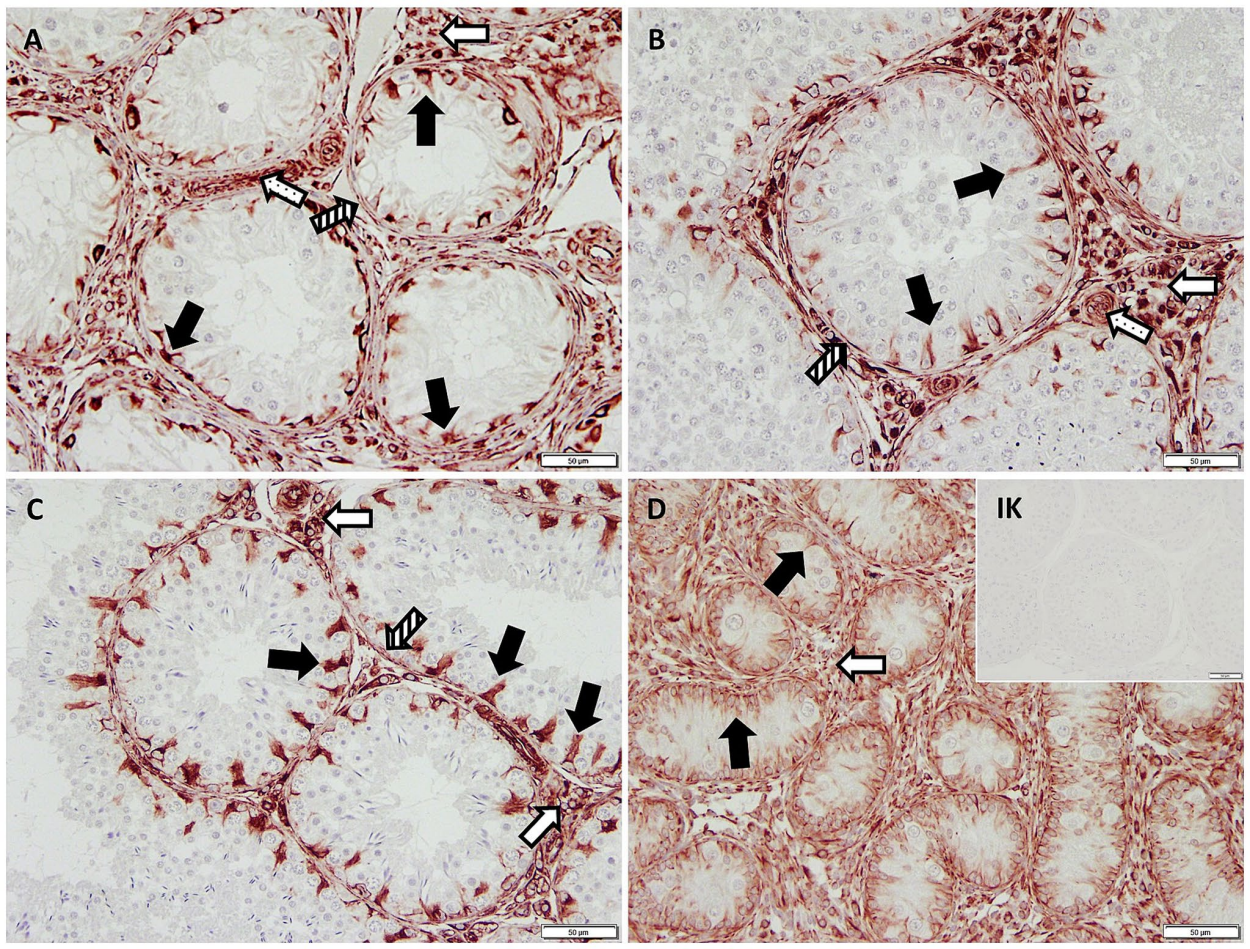
Immunopositive staining in Sertoli cell cytoplasm (black arrows) against Vimentin (200× magnification) in **(A)** early arrest CAO and **(B)** late arrest CAO, **(C)** healthy control testis, and **(D)** juvenile testis. Isotype control (IK) did not stain positive as expected. Also positive are Leydig cells (empty arrows), blood vessels (dotted arrows), and peritubular myoid cells (striped arrows).

**Figure 2:**
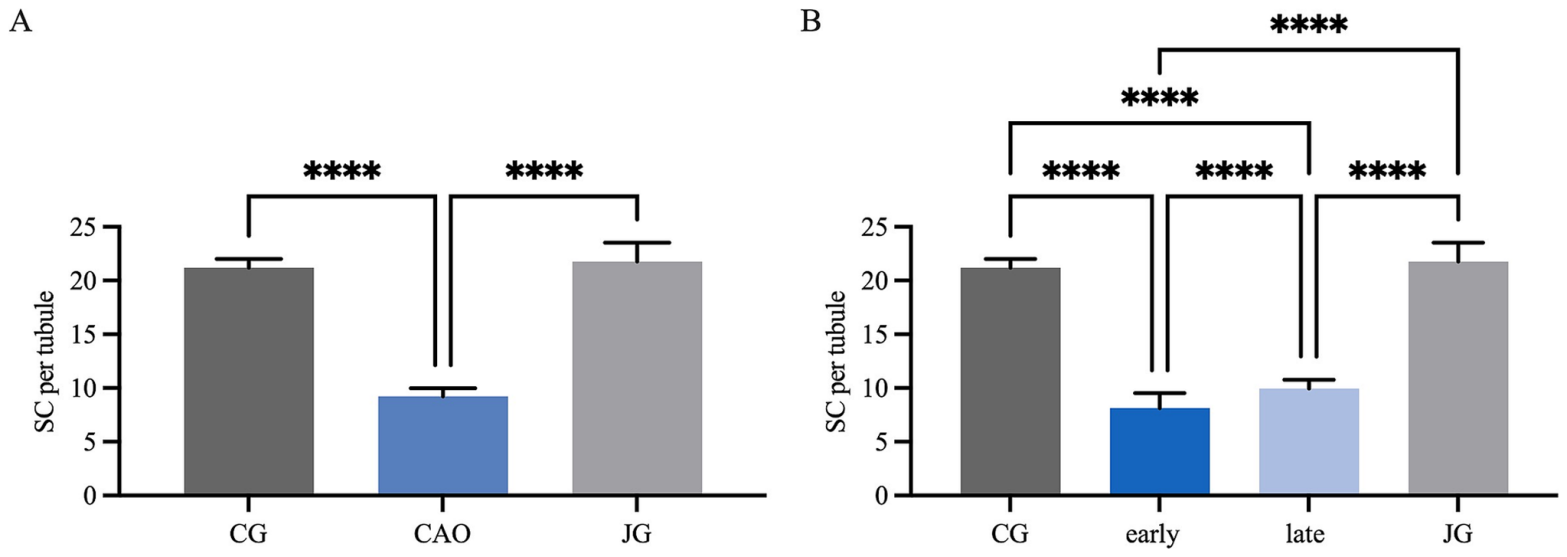
Statistical evaluation of the Sertoli cell counting based on Vimentin-immunopositive signals in Sertoli cells. The results are presented as Sertoli cells (SC) per tubule. **(A)** Control group (CG), CAO, juvenile group (JG). **(B)** Control group (CG), CAO early arrest (early), CAO late arrest (late), juvenile group (JG). All results are presented as arithmetic mean and standard deviation ( $\bar{x\;}$ ± SD). Datasets with asterisks differ significantly: each ****p < 0.0001.

IHC revealed specific immunopositive staining within the tubules against Vimentin located in the cytoplasm of SCs in CG, JG, and CAO samples (Figure 1). In addition, peritubular cells and Leydig cells stained Vimentin-positive in all groups. The number of SCs—based on Vimentin-positive immunoreactivity—differed significantly between groups (*p* < 0.0001) as displayed in Figure 2A. The number of SCs was lowest in the case of CAO with early arrest compared to all other groups (each *p* < 0.0001, Figure 2B). In addition, CAO samples with late arrest had fewer SCs than CG and JG samples (each *p* < 0.0001). However, SC numbers in CG and JG did not differ significantly.

### 3.2 AMH expression

**Figure 3:**
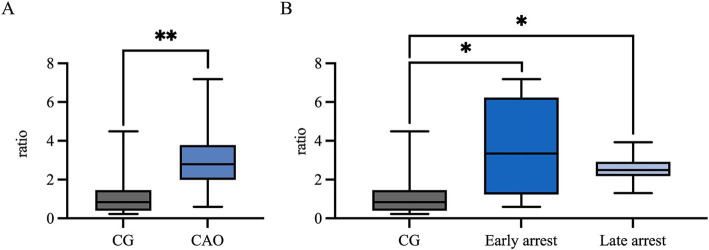
*AMH* mRNA expression (ratio) **(A)** in healthy control dogs (CG) and CAO-affected dogs, as well as **(B)** comparison of *AMH* expression in CG, CAO-affected dogs separately presented as results from early arrest and late arrest. The results are presented as Box and Whisker Plots with median, maximum, and minimum. Datasets with asterisks differ significantly: *p < 0.05, **p < 0.01.

The *AMH* mRNA expression was significantly higher in CAO than in CG (unpaired *t*-test, *p* = 0.0024, Figure 3A). Comparing ratios of CG to CAO early arrest and late arrest, an overall significant difference was identified (ANOVA, *p* = 0.0050, Figure 3B) with each group differing significantly from each other (Tukey’s multiple comparison tests, each *p* < 0.05, Figure 3B).

**Figure 4:**
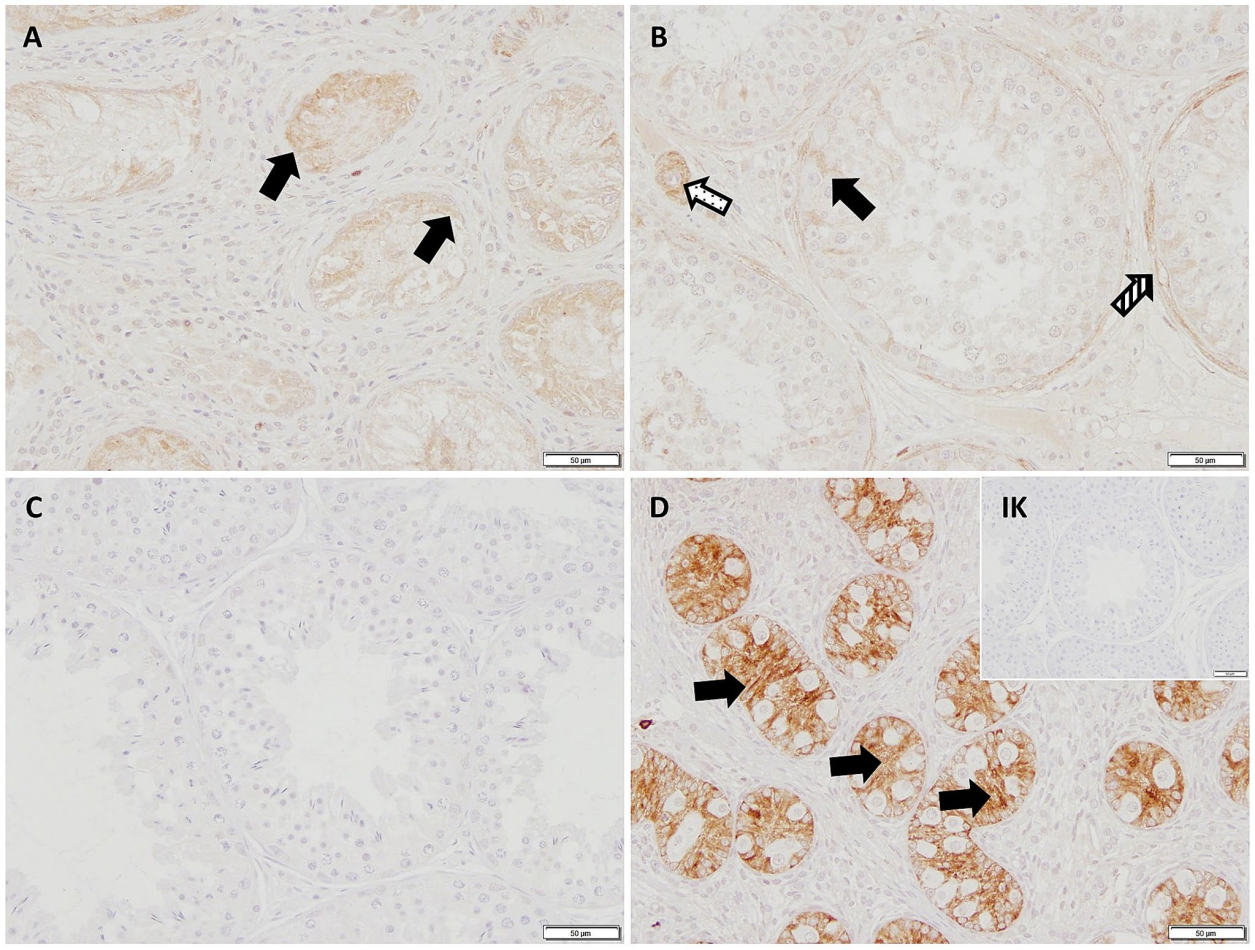
AMH-immunopositive signals (200× magnification) can be seen in the Sertoli cytoplasm (black arrows) in **(A)** early arrest CAO, **(B)** late arrest CAO, and in a juvenile dog **(D)**, whereas Sertoli cells in healthy control dogs **(C)** and in the Isotype control (IK) are negative. Peritubular myoid cells (striped arrow) and blood vessels (dotted arrow) stain immunopositively, too.

**Figure 5:**
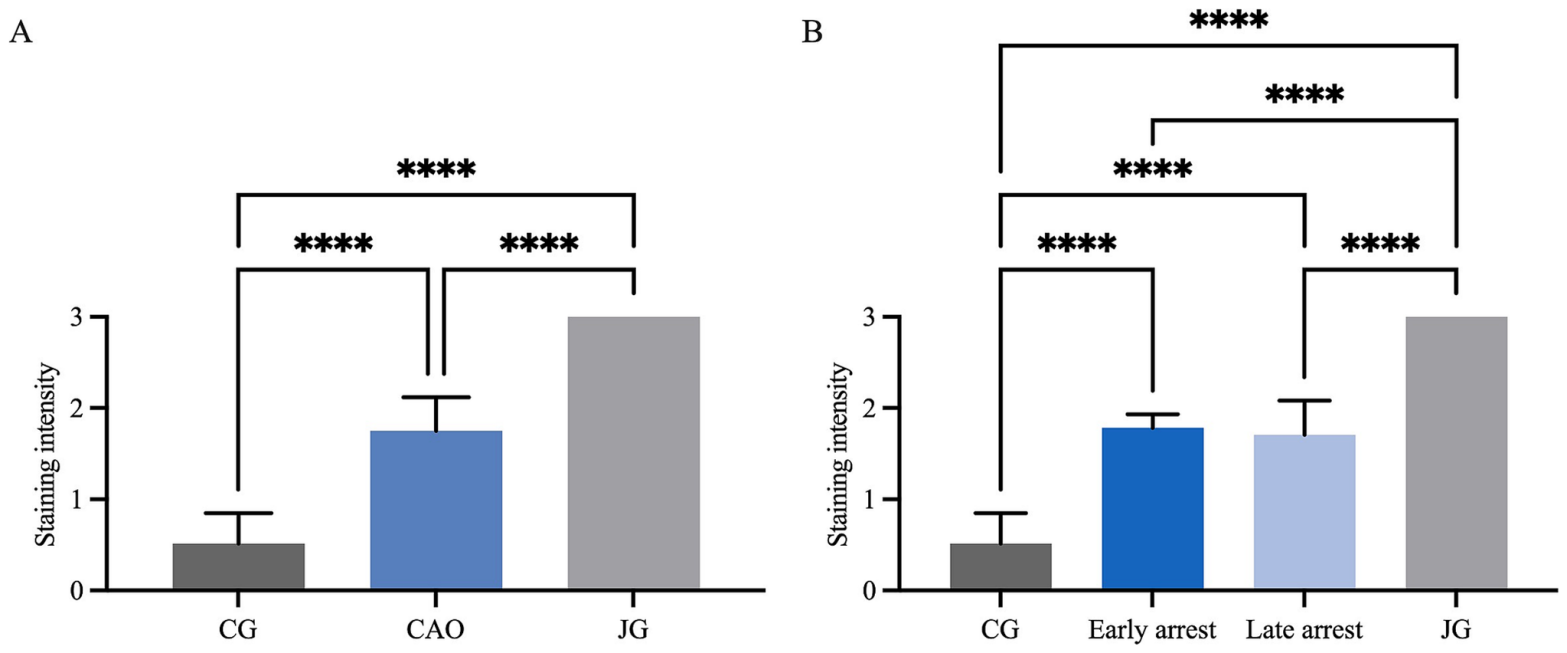
Statistical evaluation of AMH-immunopositive staining in Sertoli cells using a score range from 0 to 3 for staining intensity. Comparison between **(A)** healthy control (CG), CAO-affected and juvenile dogs (JG) and **(B)** between CG, CAO-affected dogs separately presented as results from early arrest and late arrest and juvenile dogs (JG). All results are presented as arithmetic mean and standard deviation ( $\bar{x\;}$ ± SD). Datasets with asterisks differ significantly: ****p < 0.0001.

In IHC, the cytoplasm of SCs in CAO and JG, but not in CG, stained immunopositive (Figure 4). In addition, blood vessels, occasionally, peritubular cells, and some immune cells in CAO samples stained immunopositive. Comparing the staining intensity of the SCs between groups, an overall significant difference was identified (ANOVA, *p* < 0.0001), and CG samples stained significantly less than CAO and JG samples (Tukey’s multiple comparison tests, each *p* < 0.0001, Figure 5A). Differentiating CAO samples into early arrest and late arrest and comparing their staining intensity to CG and JG, an overall significance was identified (ANOVA, *p* < 0.0001, Figure 5B). Despite the fact that all other groups differed significantly from both early arrest and late arrest (Tukey’s multiple comparison tests, each *p* < 0.0001), no significance was revealed between the early arrest and late arrest CAO dogs (Figure 5B).

**Figure 6:**
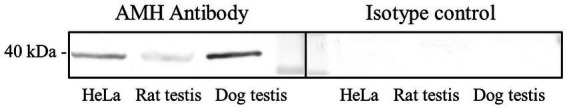
Western blot of AMH, HeLa cell lysate, and rat testis served as positive control, healthy control dog testis (CG) was tested. Protein size [in kilodalton (kDa)] is given on the left side.

Western blot was performed to confirm the specificity of the AMH antibody (Figure 6), using testicular tissue of the dog, together with rat testis and HeLa cell lysate as positive controls. The molecular weight of the canine band was equally located to the positive controls at 40 kDa. No specific immunoreactive band was visible in the negative and isotype controls from the canine testes and positive controls. AMH antibody is a 140-kDa glycoprotein composed of two identical subunits of 70 kDa (62). It occurs to have a bioreactive 30-kDa subunit, being the result of a possible second cleavage site under reducing conditions (63), which then explains our 40-kDa result as a part of the 70-kDa homodimer without the 30-kDa cleavage (64).

### 3.3 CK18 expression

**Figure 7:**
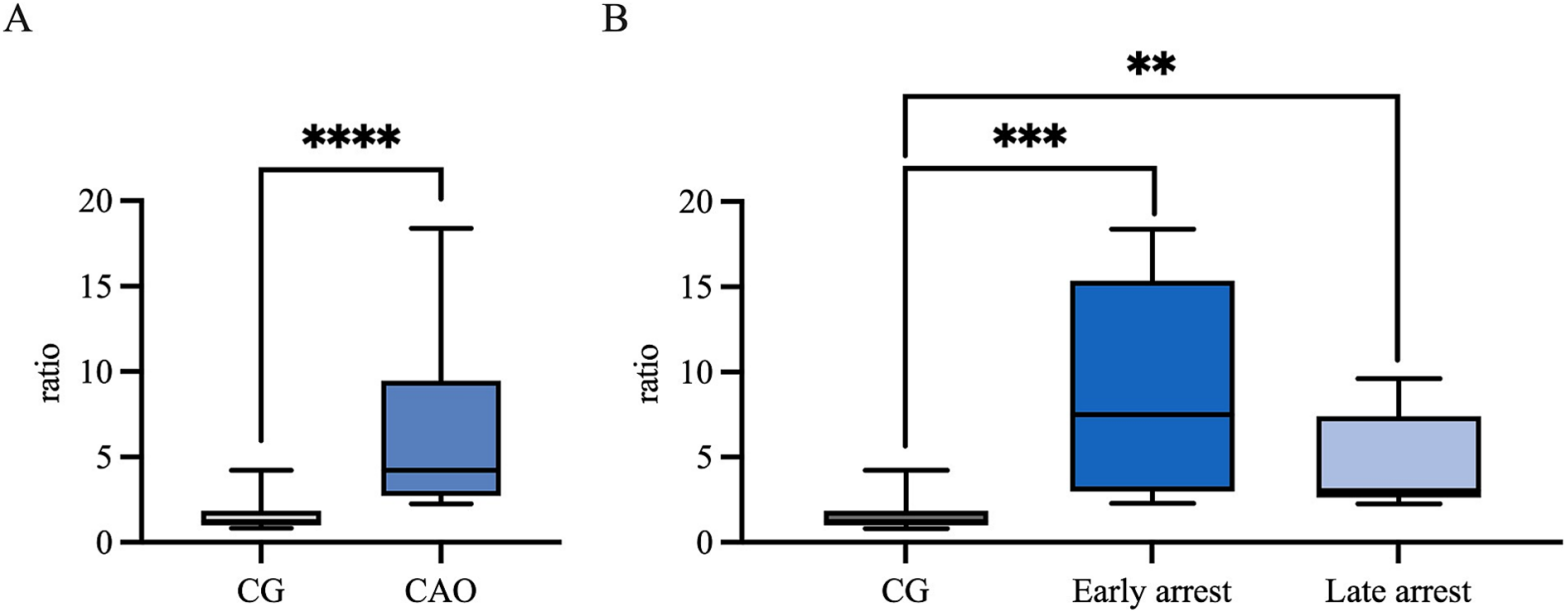
*CK18* mRNA expression (ratio) **(A)** in healthy control dogs (CG) and CAO-affected dogs, as well as **(B)** comparison of *AMH* expression in CG, CAO-affected dogs separately presented as results from early arrest and late arrest. The results are presented as Box and Whisker Plots with median, maximum, and minimum. Datasets with asterisks differ significantly: **p < 0.01, ***p < 0.001, ****p < 0.0001.

Ratios (mRNA expression) for *CK18* revealed significantly higher levels for CAO in comparison to the CG (unpaired *t*-test, *p* < 0.0001, Figure 7A). The ratios of CG to CAO early arrest and late arrest, an overall significant difference was identified (ANOVA, *p* = 0.0001, Figure 7B). The division of CAO into early arrest and late arrest compared to CG is displayed in Figure 7B (Tukey’s multiple comparison tests, each *p* < 0.05); again, no significance was revealed within the CAO group.

**Figure 8:**
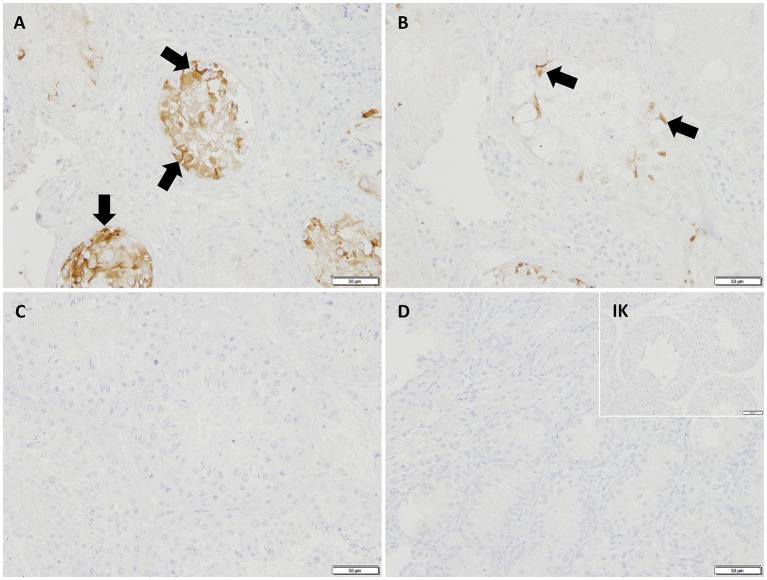
Immunopositive staining in Sertoli cell cytoplasm (black arrows) against CK18 (200 × magnification) in **(A)** early arrest CAO and **(B)** late arrest CAO, whereas Sertoli cells in **(C)** healthy control testis, **(D)** juvenile testis, and isotype control (IK) did not stain.

**Figure 9:**
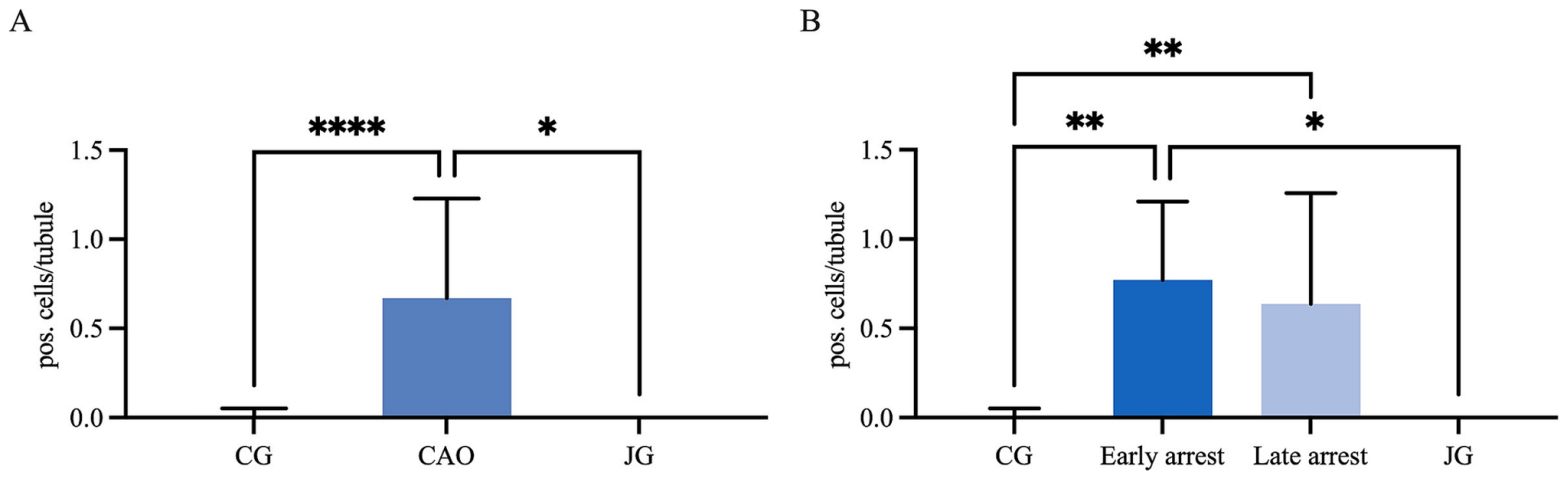
Statistical evaluation of CK18-immunopositive staining in Sertoli cells, and the results are presented as positive cells per tubule. Comparison between **(A)** healthy control (CG), CAO-affected and juvenile dogs (JG) and **(B)** between CG, CAO-affected dogs separately presented as results from early arrest and late arrest and juvenile dogs (JG). All data are presented as arithmetic mean and standard deviation ( $\bar{x\;}$ ± SD). Datasets with asterisks differ significantly: *p < 0.05, **p < 0.01, ****p < 0.0001.

In IHC, CK18 expression was restricted to the cytoplasm of SCs in CAO (Figure 8), whereas no staining was visible in SCs of CG and juvenile dogs. As expected, the number of immunopositive signals differed significantly in the overall comparison (Kruskal–Wallis test, *p* < 0.0001, Figure 9A). It was considerably lower in CG and JG compared to the CAO group (Dunn’s multiple comparison tests, *p* < 0.0001, *p* < 0.05, respectively, Figure 9A), with CG and JG not differing. Furthermore, differentiating CAO into early arrest vs. late arrest stage and comparing it to JG and CG showed an overall significance (Kruskal–Wallis test, *p* < 0.0001, Figure 9B). The significant differences revealed by Dunn’s multiple comparison tests of the early arrest stage of CAO compared to CG and JG (*p* < 0.01, *p* < 0.05) and late arrest to CG (*p* < 0.01) are shown in Figure 9B. In contrast, CAO early arrest and late arrest did not differ significantly.

**Figure 10:**
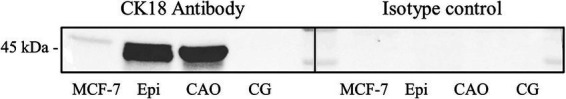
Western blot of CK18, MCF-7 cell line as a positive control, dog epididymis (Epi), CAO-affected dog testis, and healthy control dog testis (CG) were tested. Protein size [in kilodalton (kDa)] is given on the left side.

Again, to prove the specificity of the CK18 antibody by Western blot, testicular tissues of an azoospermic dog, a normospermic dog, epididymis, and an MCF-7 cell line as positive control were used (Figure 10). The molecular weight of the canine azoospermic band was equally located to the positive controls at 45 kDa. Normal, healthy dog protein revealed no signal as expected. Negative and isotype control showed no specific immunoreactive bands.

## 4 Discussion

As mentioned before, CAO is the leading cause of canine NOA (1), with NOA being the most common diagnosis in infertile male dogs (2), but also in men (3, 4, 65). Further investigations in CAO are very important as still no therapy for both species is available (66–70). The dog represents a very suitable model for humans regarding research into fertility disorders and is, therefore, moving further into the focus of the research. In particular, the similarities in the process of spermatogenesis and in the pathophysiology of various testicular diseases offer an interesting starting point for using the male dog as a model for developing therapeutic approaches to fertility disorders. The comparability of the clinical picture leads to the dog as a perfect model of CAO for human medicine, as used for environmental effects on spermatogenesis before (71, 72). Profound knowledge of CAO is essential for any therapeutic approach. As the intact somatic testicular environment is crucial for germ cell development and, more precisely, SCs are a key factor for functional spermatogenesis, reviewed by O’Donnell et al. (20), this study focused on the role of SCs in CAO. As SSCs were reduced in the CAO-affected dogs (15), we were interested if the number of SCs might be affected, too. Reduction of SCs could lead to the loss of SSCs as there occurs to be a fixed germ cell-SC ratio (22, 31, 32). A reduced number of SCs—as identified herein CAO—was likewise described in azoospermic human patients (73). Interestingly, the degree of SC reduction was correlated with the severity of disruption of spermatogenesis with fewer SCs in the early arrested CAO dogs compared to the late arrest stages. Although a causative correlation might be possible, other explanations, such as immune-mediated disruptions in the testicular microenvironment, that could independently affect both Sertoli cells and spermatogenesis, appear more probable. Future studies should aim to clarify this. The question is, what caused the reduction of SCs within the CAO? Apoptosis of SC was described to be unlikely (56); they are traditionally considered quiescent with a final number that is steady once adulthood has been reached [reviewed by Sharpe et al. (32)]. Despite apoptosis in context with SCs is mainly known in correlation with defect germ cells (56, 74, 75), apoptosis of SCs themselves was described after cypermethrin and zearalenone exposure (76, 77), and in relation to seasonal changes in hamsters (78), stallions (79), and goats (80). In humans, the number of SCs was found to be variable in individuals (22) and reduced in an age-dependent manner (31). Further research is needed to determine whether the SCs in the dog undergo apoptosis, autophagy, or similar processes and aim to understand the role of immune cells by providing mechanistic insights, e.g., by expanding on immune-mediated pathways. Our outcome is limited to the lack of data on the testicular volume. With the arrest of spermatogenesis, the size of the tubules decreases. At the same time, the interstitial compartment gets larger, caused by several histological findings such as fibrosis, immune cell infiltrations, and thickened tubular basement membranes (1). Consequently, the relation of tubular volume to interstitial compartment shifts in CAO, in addition to decreasing the total size of CAO testis. Accordingly, it would have been interesting to calculate the SC density and the number of SCs in relation to the testis volume. Unfortunately, knowledge of the numerical density of SCs in the canine testis is limited, indicating the need for further studies, taking the considerable variation regarding breed and, consequently, size and body weight into consideration. In addition, the numerical density of SC in our CAO-affected testes should be compared to age- and breed-matched normospermic control testes. Nevertheless, the total number of SCs per tubule in the CAO group compared to CG is clearly reduced, which might be associated with the loss of SSCs—either as a cause or consequence.

In addition, the fact that the SCs were previously described as stable in number after reaching adulthood, the developmental status was also considered to be fixed (32). Typically, SCs develop through puberty due to testosterone influence into their mature but quiescent state (41, 43, 81). Whereas formerly SCs had been considered as postmitotic, terminally differentiated cells, *in vitro* studies on mouse and human SCs revealed possible further differentiation, indicating that adult SCs more resemble arrested proliferating cells (82). In humans, *AMH* appears to be expressed until germ cells enter meiosis, and androgen receptor signaling starts at puberty (83). On the other hand, CK18 is a fetal marker, and its expression vanishes after the 20th gestational week (27, 38). As no comparable data on the expression of CK18 in the juvenile dog are available to date, we aimed and confirmed absent immunopositive staining in the juvenile dogs at 8 weeks of age. There have been few studies on maturation status with re-expression of immaturity SC markers in dogs with deslorelin implant (46), cryptorchism (50), atrophic testes (42), and under neoplastic conditions (33, 34, 44), but research about CAO is still lacking. As CAO-affected dogs had been previously fertile and healthy canine SCs do not express AMH and CK 18, it appears probable that SCs in now CAO-affected dogs might have been devoid of expression earlier. Consequently, the confirmed expression of AMH and CK18 at mRNA and protein levels in the CAO-affected dogs might be considered as a re-expression. This leads to the conclusion that SCs (at least partly) return to an immature(−like) state. This effect in NOA (43, 84) and SCO (43, 47, 49, 85) affected men has already been described, which underlines the value of the dog as a model for human research. Interestingly, we had a constant expression of AMH in nearly all SCs, while CK18 only occurred in a few SCs or all SCs in some tubules. Some studies mention an association between the expression of AMH and possible tumor development. In dogs, however, only geriatric dogs and cryptorchids have so far been described as having a risk of developing unilateral SC tumors, which argues against an increased risk in the CAO group, especially as the testicles are equally affected on both sides (86, 87). All dogs included in our study had normally descended testes and no significant difference in terms of AMH expression between both testes. In addition, dogs with SC tumors tend to have high serum estradiol levels and low testosterone concentrations (88), which cannot be confirmed in our previous study of CAO dogs (1). Concluding that, CAO appears not to be connected to a higher risk of tumor development. Regarding our results of CK18, higher expression in SCO tubules and more damaged tissue could be observed, which might be associated with the fact that it is only expressed in very early maturation states of (fetal) SCs in contrast to AMH, which is expressed until puberty. Consequently, we further hypothesize that the degree of functional disturbance of the individual SCs appears to be linked to their expression of immaturity markers. A recent *in vitro* study about the impact of follicle-stimulating hormone (FSH) on pre-pubertal porcine SC revealed some interesting alterations in the expression of AMH and Inhibin B (89), proposing FSH as a treatment option for immature SC. Another study investigating the effect of co-culturing human NOA patient-derived SCs and germ cells confirmed a beneficial effect of FSH and testosterone supplementation in the culture medium on germ cell maturation (90). These authors postulated that the beneficial effects of FSH were related to restoring SC-derived cytokine expressions that were previously altered due to NOA, thereby impairing spermatogenesis (90). Studies on cytokine expression in canine NOA or even canine CAO are still missing and urgently needed to prove FSH’s benefits in affected dogs. In addition, different SC markers such as Wnt oncogene analog 5 (WNT5) (91, 92) and bone morphogenetic protein 4 (BMP4) (93) are also known to be altered in immature SC, what would be interesting to investigate in the CAO-affected testis too, because high levels of WNT5, for example, appear to inhibit SC maturation (30) and are connected to immune cell infiltrations (94). Despite this, the exact reason for the SC dedifferentiation remains unknown. It appears to be connected to the disruption of the BTB, demonstrated by a study with Connexin43 (CX43)-knockout mice, where the SCs showed a pre-pubertal phenotype (95). Interestingly, our group identified an altered expression of BTB proteins in a collective of canine CAO-affected testis (96).

Nevertheless, immature, dedifferentiated SCs will not be able to support the spermatogonial stem cells properly for functioning spermatogenesis; thus, stem cell-based therapeutic options mentioned by Reifarth et al. (15) should relate to optimizing the stem cell niche, especially regarding the SC number and function.

## 5 Conclusion

Our study clearly shows that in addition to the earlier described germ cells, SCs, as crucial part of the testicular stem cell niche, are affected by CAO in the dog. They were reduced in number and re-expressed the immaturity markers AMH and CK18 at mRNA and protein levels. This might be linked to the loss of stem cells and may be related to the immune cell infiltration, either directly or indirectly by, e.g., cytokine secretion and inflammatory conditions. As SCs are essential for functional spermatogenesis, further research, especially in understanding the causes of dedifferentiation, into the reinitiating the maturation process and gaining deeper insights into the microenvironmental effects of SCs in CAO is requested to approach possible therapeutic approaches.

## Data Availability

The datasets presented in this study can be found in online repositories. The names of the repository/repositories and accession number(s) can be found in the article/supplementary material.

## Footnotes

^1^ https://blast.ncbi.nlm.nih.gov/Blast.cgi
